# Synthesis and Evaluation of Neuroprotective Selenoflavanones

**DOI:** 10.3390/ijms161226188

**Published:** 2015-12-10

**Authors:** Yong-Sung Choi, Dong-Myung Kim, Yoon-Jung Kim, Sai Yang, Kyung-Tae Lee, Jong Hoon Ryu, Jin-Hyun Jeong

**Affiliations:** 1 College of Pharmacy, Yonsei Institute of Pharmaceutical Sciences, Yonsei University, 85 Songdogwahak-ro, Yeonsu-gu, Incheon 21983, Korea; uriys2@gmail.com (Y.-S.C.); sunshine333@naver.com (Y.-J.K.); saiyang871001@gmail.com (S.Y.); 2 Office of Research Affairs, Yonsei University, 50 Yonsei-ro, Seodaemun-gu, Seoul 03720, Korea; dongmk@yonsei.ac.kr; 3 College of Pharmacy, Kyung Hee University, 26 Kyungheedae-ro, Dongdaemun-gu, Seoul 02453, Korea; ktlee@khu.ac.kr (K.-T.L.); jhryu63@khu.ac.kr (J.-H.R.)

**Keywords:** selenoflavanone, flavanone, selenium, neuroprotect, antioxidant, blood-brain barrier (BBB)

## Abstract

The physicochemical properties and antioxidant activity of a molecule could be improved by the substitution of an oxygen atom in a molecule with selenium. We synthesized selenoflavanones and flavanones to evaluate their neuroprotective effects. The selenoflavanones showed improved physicochemical properties, suggestive of the ability to pass through the blood-brain barrier (BBB). They showed *in vitro* antioxidant effects against hydrogen peroxide, and did not result in severe cytotoxicity. Moreover, infarction volumes in a transient ischemia mouse model were significantly reduced by the selenoflavanone treatments.

## 1. Introduction

Flavonoids, a group of phenolic compounds with diverse chemical structures and characteristics, occur naturally in the fruits, seeds, flowers, and bark of plants [1,2]. To date, several studies have demonstrated the anti-oxidant, anti-inflammation, and anti-cancer activities of flavonoids [3,4,5]. Reactive oxygen species (ROS), such as hydrogen peroxide, are generated by cerebral ischemia conditions, such as stroke [6,7]. These ROS induce cell apoptosis, resulting in damage to brain cells [8,9,10]. Therefore, an antioxidant compound that scavenges ROS could play an important role in the treatment of neurodegenerative disease. The neuroprotective effects of antioxidants have been well studied [11,12]. Recently, the flavonoids were highlighted as potentially promising neuroprotective agents [13,14,15], but their high polarity limits their ability to penetrate the blood-brain barrier (BBB).

Our group recently reported that the substitution of an oxygen atom in flavone with selenium led to improvement in the physicochemical properties and biological activity of molecules [16]. These selenoflavones had lower polarity (tPSA) and higher lipophilicity (Clog*P*) than their corresponding flavones, and, consequently, were anticipated to better penetrate the BBB. Moreover, they showed improved *in vitro* antioxidant activity with respect to scavenging hydrogen peroxide. Therefore, we expected the selenoflavonoids to be more druggable and more potent neuroprotective agents.

## 2. Results and Discussion

**Figure 1:**
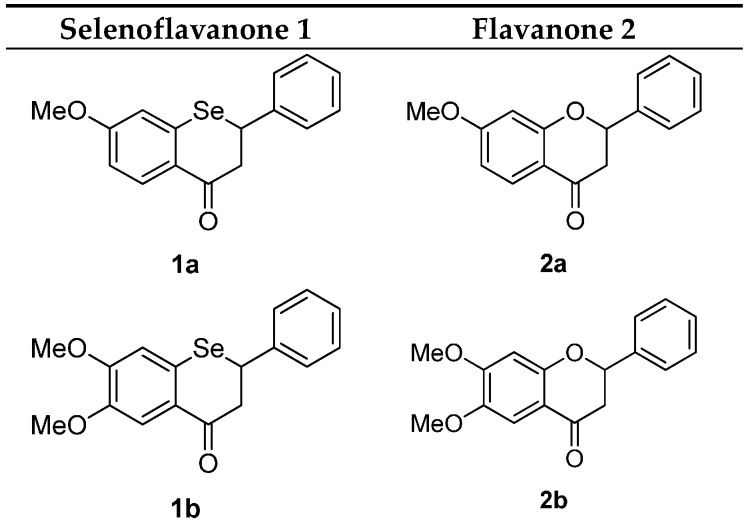
The structures of selenoflavanones and flavanones.

Based on previous studies, we decided to synthesize some additional selenoflavanoids and evaluate their *in vivo* neuroprotective activity. We designed and synthetized two simple selenoflavanones (**1a** and **1b**). The corresponding flavanones (**2a** and **2b**) were also synthesized to allow for a comparison of their neuroprotective activities. The structures of the compounds utilized in this study are indicated in Figure 1.

### 2.1. Synthesis of the Selenoflavanones and Flavanones

**Scheme 1:**
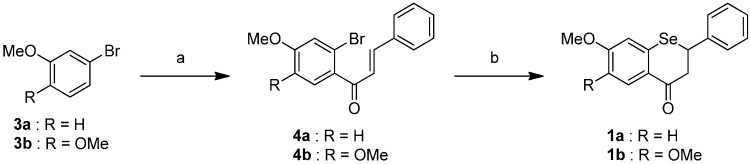
Reagents and conditions: (**a**) cinnamoyl chloride, AlCl_3_, CH_2_Cl_2_, 0 °C, 2 h; (**b**) *t*-BuLi, Se, THF, −78 °C to room temperature, 75 min.

The synthetic pathway of the Selenium flavanones **1** is described in Scheme 1. Compound **4** was conducted from bromobenzene **3** and cinnamoyl chloride under AlCl_3_ conditions [17]. To introduce a selenium atom into the heterocycle, compound **4** was treated with *t*-BuLi, followed by elemental selenium [18]. The selenoflavanones **1** were obtained using a two-step synthesis resulting in 76%–80% overall yields.

**Scheme 2:**
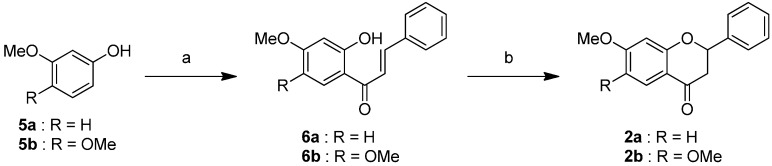
Reagents and conditions: (**a**) cinnamoyl chloride, BF_3_-Et_2_O, reflux, 15 min; (**b**) NaOAc, EtOH, H_2_O (cat.), reflux, 6 h.

We successfully synthesized the corresponding flavanones 2, as shown in Scheme 2. Acylation of the phenol compound **5** with cinnamoyl chloride was carried out under BF_3_-Et_2_O conditions [19]. Treatment of compound **6** with NaOAc led to intramolecular cyclization to form the flavones **2** [20] in 72%–75% two-step yields.

### 2.2. Physicochemical Properties of the Selenoflavanones and Flavanones

First, we evaluated the physicochemical properties of the selenoflavanones and flavanones. Polar surface area (PSA) is one of the drug-like factors demonstrating the polarity of molecules. The partition coefficient (log*P*) is known to be a measure of lipophilicity. The topological polar surface area (tPSA) and calculated partition coefficient (Clog*P*) were generated using Cambridgesoft ChemBioDraw software (Waltham, MA, USA) (Table 1). The selenoflavanones had lower tPSA and higher Clog*P* values than their corresponding flavanones. By substituting oxygen with selenium, the polarity and lipophilicity were improved, likely allowing the compounds to more easily pass through the BBB [21,22].

**Table 1:** Calculated physicochemical properties of selenoflavanones and flavanones.

Selenoflavanone	Physicochemical Properties		Flavanone	Physicochemical Properties	
	tPSA ^a^ (Å^2^)	Clog*P* ^a^		tPSA ^a^ (Å^2^)	Clog*P* ^a^
1a	26.3	4.20	2a	35.5	3.50
1b	35.5	3.88	2b	44.8	3.11

^a^ Generated by Cambridgesoft ChemBioDraw software.

### 2.3. Biological Activities of the Selenoflavanones and Flavanones

#### 2.3.1. Antioxidant Effect and Cytotoxicity of the Selenoflavanones and Flavanones

**Figure 2:**
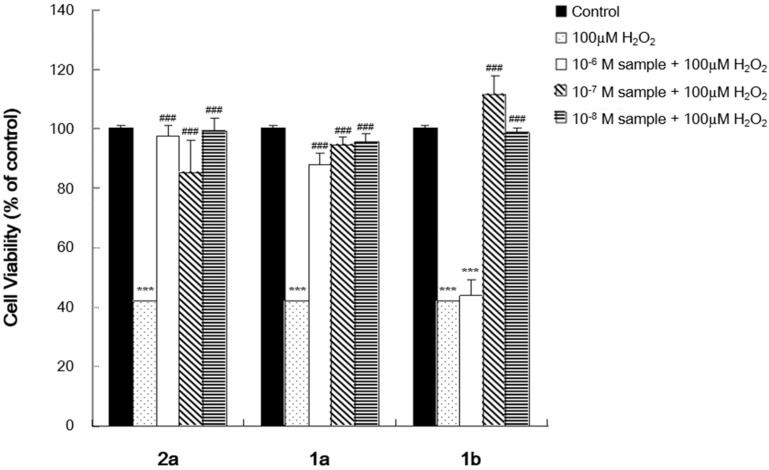
Changes in cell viability due to treatment with compounds. *** *p* < 0.001 compared with the control group. ^###^ *p* < 0.001 compared with the H_2_O_2_-only treated group.

To confirm the antioxidant effect and cytotoxicity of the compounds, cell viability in the presence of hydrogen peroxide (100 µM) was measured using an MTT assay (tetrazolium dye colorimetric assay) [23] (Figure 2). Treatment with hydrogen peroxide (H_2_O_2_) caused oxidative stress and resulted in the death of 60% of SH-SY5Y cells. H_2_O_2_ induced cell death could be reduced by antioxidant–reactive oxygen species scavenger. All of the selenoflavanone (**1a**, **1b**) and flavanone (**2a**) treatment groups showed fully recovered cell viability at all concentrations, except for compound **1b** (10^−6^ M). Based on these observations, we believe that flavanone **2a** and the selenoflavanones have antioxidant activity, and that selenoflavanone 1b behaves as a pro-oxidant at high concentrations, similar to some flavonoids, which show antioxidant or pro-oxidant effects depending on their concentration [8,24]. The selenoflavanones did not result in cytotoxicity at low concentrations. Consequently, we believe that they could be developed into neuroprotective drugs.

#### 2.3.2. Neuroprotective Activity of the Selenoflavanones and Flavanones

**Figure 3:**
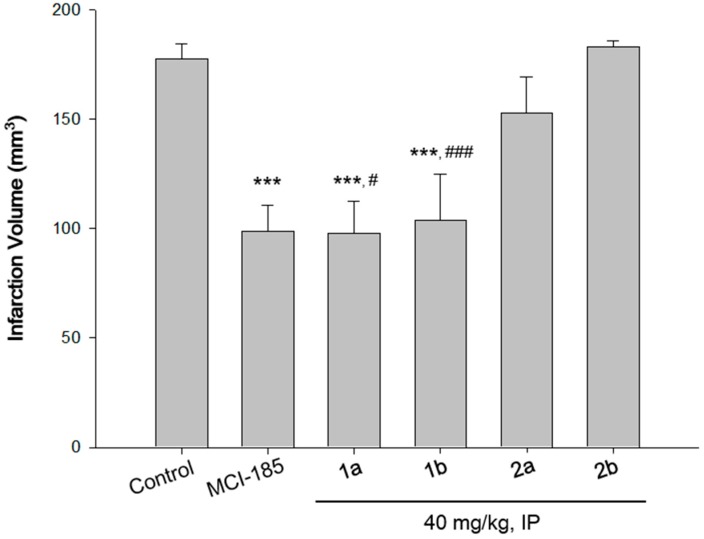
Changes in infarction volume due to treatment with compounds. MCI-185: positive control (5 mg/kg, iv); **1a**: 7-methoxylselenoflavanone; **2a**: 6,7-dimethoxyselenoflavanones, **2a**: 7-methoxyflavanone; **2b**: 6,7-dimethoxyflavanone; IP: intraperitoneal; *** *p* < 0.001 compared with the control group. ^#^ *p* < 0.05, ^###^ *p* < 0.001 compared with the flavanone treated group.

Next, we studied the *in vivo* neuroprotective activity of the compounds. A transient ischemia mouse model [25] was used to confirm the actual changes in infarction volume resulting from treatment with the compounds (Figure 3). Considerable and reproducible focal infarction and hemispheric bulging were induced all over the cortical and subcortical structures when the subjects were treated under middle cerebral artery occlusion (MCAO) for 90 min followed by 22.5 h of reperfusion condition. An extensive infarct was observed in the control group animals, as shown in Figure 3 (the total infarction volume was 177.58 ± 6.69 mm^3^). In comparison with the control group, the anterior and medial striatum, and large areas of the cortex were remained unaffected in the selenoflavanone-treated group. In other words, selenoflavanone treatment significantly reduced the total infarction volumes in the ipsilateral hemisphere of the ischemia-reperfusion mice. Total infarct volumes at doses of 40 mg/kg were reduced by approximately 45% or 41% by either 7-methoxyselenoflavanone (**1a**) or 6,7-dimethoxyselenoflavanone (**1b**) treatment, respectively. The selenoflavanones showed better protective effects than the flavanones. Moreover, treatment with MCI-186 (positive control, 5 mg/kg, intravenous (IV)) reduced the infarct volume by 45%. Selenoflavanones (**1a**, **1b**) showed more potent neuroprotective activity than corresponding flavanones (**2a**, **2b**).

Blood concentration and brain level of the compounds were not monitored. Nevertheless, changes in physicochemical properties suggested that selenium substitution improved bioavailability and/or BBB penetration of the compound. Flavanones also showed similar *in vitro* antioxidant activity, but it had more polar and hydrophilic characters than corresponding selenoflavanones. As a result, **1a** and **1b** proved to have more potential to penetrate membranes in gastrointestinal or brain while **2a** and **2b** not. Antioxidant or pro-oxidant activity of compounds can be switched by concentration and other conditions [8,24]. Although oxidation potential of selenoflavonoids has not been identified yet, molecular behavior between antioxidant and pro-oxidant might be affected by the substituted atom. We postulate that the results of the neuroprotective activity assay were affected by changes in both the physicochemical properties and oxidation-potential of the compounds as a result of the selenium substitution.

## 3. Experimental Section

### 3.1. Chemistry

#### 3.1.1. General Information

CH_2_Cl_2_ and THF used for the reaction were Liquid Chromatography grade reagents and were redistilled from calcium hydride and sodium benzophenone ketyl, respectively. All other reagents were obtained commercially and used as received without additional purification. Reactions were monitored by thin-layer chromatography on 0.25 mm silica plates (F-254) visualized with ultraviolet (UV) light (254 nm) and KMnO_4_ solution. Flash chromatography was performed using silica gel (230–400 mesh) with hexanes and EtOAc as eluents. ^1^H and ^13^C nuclear magnetic resonance (NMR) spectra were recorded on Varian Gemini 200 MHz or Agilent 400 MHz NMR spectrometers. Chemical shifts (δ) are reported in parts per million (ppm), and coupling constants (*J*) are expressed in Hertz (Hz).

#### 3.1.2. General Procedure for the Acylation of Bromobenzenes

Aluminum chloride powder (2.0 mmol) was suspended in CH_2_Cl_2_ (10 mL) at 0 °C. Cinnamoyl chloride (1.2 mmol) in CH_2_Cl_2_ (5 mL) was added and the mixture was stirred for 30 min. Then, a solution of bromobenzene (1.0 mmol) in CH_2_Cl_2_ (5 mL) was added dropwise and stirred for another 2 h. After the addition of water (20 mL), the organic layer was separated and the aqueous layer was extracted with CH_2_Cl_2_ (3 × 30 mL). The combined organic extracts were filtered through a celite pad, washed with water (3 × 100 mL) and brine (100 mL), dried (MgSO_4_), and concentrated. The resulting residue was purified by flash chromatography to give (**4a**, **4b**) in 85%–87% yields.

(*E*)-1-(2-Bromo-4-methoxyphenyl)-3-phenylprop-2-en-1-one (**4a**) ^1^H NMR (400 MHz, CDCl_3_) δ 7.60–7.55 (m, 2H), 7.54–7.45 (m, 2H), 7.43–7.36 (m, 3H), 7.22–7.14 (m, 2H), 6.92 (dd, *J* = 8.6, 2.4 Hz, 1H), 3.86 (s, 3H); ^13^C NMR (100 MHz, CDCl_3_) δ 193.4, 161.7, 145.4, 134.8, 133.5, 131.3, 130.8, 129.1, 128.6, 126.2, 121.2, 119.1, 113.4, 55.9.

(*E*)-1-(2-Bromo-4,5-dimethoxyphenyl)-3-phenylprop-2-en-1-one (**4b**) ^1^H NMR (400 MHz, CDCl_3_) δ 7.65–7.58 (m, 2H), 7.55 (d, *J* = 16.0 Hz, 1H), 7.46–7.37 (m, 3H), 7.22 (d, *J* = 15.9 Hz, 1H), 7.08 (s, 1H), 7.04 (s, 1H), 3.94 (s, 3H), 3.90 (s, 3H); ^13^C NMR (100 MHz, CDCl_3_) δ 193.3, 151.2, 148.3, 145.2, 134.6, 133.2, 130.7, 129.0, 128.6, 125.9, 115.9, 112.4, 111.2, 56.3, 56.2.

#### 3.1.3. General Procedure for the Formation of Selenoflavanones

To a THF (10 mL) solution of (**4a**, **4b**) at −78 °C, 1.7 M *t*-BuLi solution in pentane was slowly added over a 10 min period. The reaction mixture was stirred at 0 °C for 30 min, and selenium powder (0.34 mmol) was added in two portions. The reaction mixture was kept at 0 °C for 5 min and then at room temperature for 30 min. After the addition of HCl (1 M, 10 mL), the organic material was extracted with Et_2_O (3 × 10 mL). The combined organic extracts were dried over MgSO_4_ and concentrated. The residue was dissolved in absolute EtOH (10 mL) and stirred vigorously in the presence of NaOH (15 mg) for 30 min. The solution was concentrated, and the resulting residue was purified by flash chromatography to give (**1a**, **1b**) in 88%–92% yields.

7-Methoxyselenoflavanone (**1a**) ^1^H NMR (200MHz, CDCl_3_) *δ* 7.16 (m, 3H), 7.05 (m, 1H), 6.92 (d, *J* = 8.8 Hz, 1H), 6.71 (dd, *J* = 11.0, 2.4 Hz, 1H), 3.8 (s, 3H), 3.42 (m, 2H), 3.05 (dd, *J* = 16.1, 5.3 Hz, 1H); ^13^C NMR (50 MHz, CDCl_3_) *δ* 202.9, 161.4, 141.6, 134.1, 130.8, 129.6, 127.7, 126.3, 118.8, 113.1, 55.8, 52.2, 43.8.

6,7-Dimethoxyselenoflavanone (**1b**) ^1^H NMR (200MHz, CDCl_3_) *δ* 7.14 (m, 3H), 7.02 (m, 2H), 6.95 (s, 1H), 6.12 (s, 1H), 3.89 (s, 3H), 3.61 (s, 3H), 3.46 (m, 2H), 2.98 (dd, *J* = 15.6, 5.0 Hz, 1H); ^13^C NMR (50 MHz, CDCl_3_) *δ* 204.3, 150.6, 147.8, 141.3, 134.2, 129.7, 127.5, 126.2, 115.3, 111.8, 109.4, 56.2, 55.8, 52.7, 43.9.

#### 3.1.4. General Procedure for the Acylation of Phenols

An appropriate phenol compound (1 mmol) and cinnamoyl chloride (1.07 mmol) were dissolved in a BF_3_-Et_2_O complex (5 mL) and heated to reflux. After a 15 min reaction, the reaction mixture was quenched with an excess of water. Then, the organic layer was separated and the aqueous layer was extracted with EtOAc (2 × 10 mL). The combined organic extracts were washed with water (2 × 10 mL) and brine (10 mL), dried (MgSO_4_), and concentrated. The resulting residue was purified by flash chromatography to give (**6a**, **6b**) in 84%–90% yields.

(*E*)-1-(2-Hydroxy-4-methoxyphenyl)-3-phenylprop-2-en-1-one (**6a**) ^1^H NMR (400 MHz, CDCl_3_) δ 13.44 (s, 1H), 7.90 (d, *J* = 15.5 Hz, 1H), 7.84 (d, *J* = 8.6 Hz, 1H), 7.68–7.64 (m, 2H), 7.59 (d, *J* = 15.5 Hz, 1H), 7.46–7.40 (m, 3H), 6.55–6.44 (m, 2H), 3.87 (s, 3H); ^13^C NMR (100 MHz, CDCl_3_) δ 191.9, 166.7, 166.2, 144.4, 134.8, 131.2, 130.7, 129.0, 128.5, 120.3, 114.1, 107.8, 101.1, 55.6.

(*E*)-1-(2-Hydroxy-4,5-dimethoxyphenyl)-3-phenylprop-2-en-1-one (**6b**) ^1^H NMR (400 MHz, CDCl_3_) δ 8.20 (d, *J* = 15.2 Hz, 1H), 7.70 (d, *J* = 7.2 Hz, 2H), 7.58–7.45 (m, 3H), 7.39 (d, *J* = 15.2 Hz, 1H), 7.04 (s, 1H), 6.40 (s, 1H), 5.30 (s, 1H), 3.98 (s, 3H), 3.92 (s, 3H); ^13^C NMR (100 MHz, CDCl_3_) δ 180.8, 165.2, 163.7, 150.8, 144.7, 133.9, 132.7, 129.8, 129.3, 115.7, 109.5, 106.5, 101.8, 57.0, 56.5.

#### 3.1.5. General Procedure for the Formation of Flavanones

To a solution of (**6a**, **6b**) (0.1 mmol) and sodium acetate (50 mg) in EtOH (5 mL), 1 drop of water was added and the mixture was heated to reflux for 6 h. The reaction mixture was poured into cold water and extracted with EtOAc (2 × 10 mL). The combined organic extracts were washed with water (2 × 10 mL) and brine (10 mL), dried (MgSO_4_), and concentrated. The resulting residue was purified by flash chromatography to give (**2a**, **2b**) in 80%–89% yields.

7-Methoxyflavanone (**2a**) ^1^H NMR (400 MHz, CDCl_3_) δ 7.88 (d, *J* = 8.8 Hz, 1H), 7.52–7.36 (m, 5H), 6.63 (dd, *J* = 8.8, 2.3 Hz, 1H), 6.51 (d, *J* = 2.3 Hz, 1H), 5.48 (dd, *J* = 13.3, 2.8 Hz, 1H), 3.84 (s, 3H), 3.05 (dd, *J* = 16.9, 13.3 Hz, 1H), 2.84 (dd, *J* = 16.9, 2.9 Hz, 1H); ^13^C NMR (100 MHz, CDCl_3_) δ 190.6, 166.2, 163.5, 138.8, 128.8, 128.7, 126.1, 114.8, 110.3, 100.9, 80.0, 55.6, 44.3.

6,7-Dimethoxyflavanone (**2b**) ^1^H NMR (400 MHz, CDCl_3_) δ 7.54–7.37 (m, 5H), 7.33 (s, 1H), 6.54 (s, 1H), 5.47 (dd, *J* = 13.5, 2.9 Hz, 1H), 3.91 (s, 3H), 3.90 (s, 3H), 3.05 (dd, *J* = 16.9, 13.5 Hz, 1H), 2.83 (dd, *J* = 16.9, 3.0 Hz, 1H); ^13^C NMR (100 MHz, CDCl_3_) δ 190.7, 158.0, 156.3, 144.7, 138.8, 128.9, 128.8, 126.2, 113.2, 106.6, 100.3, 80.3, 56.3, 56.2, 44.2.

### 3.2. In Vitro Cell Viability

#### 3.2.1. Cell Culture

SH-SY5Y cells (human neuroblastoma) were obtained from the Korean cell line bank (KCLB, Seoul, Korea). Cells were cultured in minimum essential medium (MEM) supplemented with 10% heat-inactivated fetal bovine serum (FBS), penicillin (100 U/mL) and streptomycin sulfate (100 µg/mL) (Life Technologies, Grand Island, NY, USA). Cells were cultured at 37 °C in an atmosphere of 5% CO_2_.

#### 3.2.2. MTT Assay

SH-SY5Y cells (1 × 10^4^) were seeded in wells containing 100 µL of Roswell Park Memorial Institute (RPMI) medium supplemented with 10% FBS in a 96-well plate. Cells were pretreated with various concentrations (10^−6^–10^−8^ M) of the test compounds for 1 h, followed by exposure to 100 µM of H_2_O_2_ for another 3 h. To produce oxidative stress, H_2_O_2_ was freshly prepared from a 30% stock solution prior to each experiment. The control cells were added to the same medium without H_2_O_2_ or test compounds. Then media were carefully changed to new media (H_2_O_2_ free). After 48 h incubation, 50 µL of MTT (5 mg/mL stock solution) was added and the plates were incubated for an additional 4 h. The medium was discarded and the formazan blue, which was formed in the cells, was dissolved with 100 µL DMSO. The optical density was measured at 540 nm. Data were expressed as mean ± S.E.M. Statistical analyses were performed using one-way analysis of variance (ANOVA) followed by the Newman-Keuls test. *** *p* < 0.001 compared with the control group. ^###^ *p* < 0.001 compared with the H_2_O_2_-only treated group

### 3.3. In Vivo Neuroprotective Activity

#### 3.3.1. Transient Ischemia Model

All *in vivo* experimental sections were carried out in accordance with the Principles of Laboratory Animal Care (National Institutes of Health (NIH) publication (NIH: Bethesda, MD, USA), #85-23, revised 1985) and the Animal Care and Use guidelines of Kyung Hee University, Korea. All test subjects were adult male imprinting control region (ICR) mice weighing between 24–32 g at the time of surgery. Anesthesia of the animals was conducted in a chamber with a mixture of N_2_O and O_2_ (70:30) containing 2.5% isoflurane, and were continued by inhalation of 1.5% isoflurane. Intraluminal filament method [25] with the modifications described previously [26] was used to induce middle cerebral artery occlusion (MCAO). The procedures were as in the following. A mid-line incision was made on the ventral surface of the neck, and the right common carotid arteries and external carotid artery were isolated and ligated with an 8.0 silk suture. A polyamide monofilament (Ethilon, Johnson and Johnson Intl., Zaventum, Belgium) coated into a round tip with silicone resin (Xantopren, Byer Dental, Kiel, Germany) was introduced into the intracranial internal carotid artery through an incision in the common carotid artery. The filament was then carefully advanced approximately 10 mm distal to the carotid bifurcation, beyond the origin of the middle cerebral artery. After 90 min of MCAO, mice were re-anesthetized and the occluding filament was gently withdrawn back into the common carotid artery to allow reperfusion to take place. Sham-operated mice underwent the same surgical procedure, except that the filament was not advanced far enough to occlude the middle cerebral artery. Body temperature was maintained at 37 ± 0.5 °C throughout the surgery using a heating pad (Biomed S.L., Balearic Islands, Spain). Heart rate, arterial blood oxygen saturation of arterial hemoglobin and ECG were monitored throughout the procedure (SurgiVet, Norwell, MA, USA).

#### 3.3.2. Drug Administration

Mice subjected to MCAO were randomly assigned to receive either drug or vehicle treatments. Each drug (40 mg/kg), dissolved in 6% Cremophor with 0.5% DMSO, was introduced intraperitoneally at 30 min after MCAO. MCI-186 (3-Methyl-1-phenyl-2-pyrazolin-5-one, Sigma-Aldrich Co. Natick, MA, USA) was used as a positive control. MCI-186 is a free radical scavenger that has been used to reduce the neuronal damage following acute brain ischemia and subsequent cerebral infarction [27]. It is also called Edaravone and has marteked in Japan by the brand name Radicut. To the mice in the MCI-186 treatment group, 5 mg/kg of MCI-186 dissolved in saline was injected intravenously using the same time schedule. The vehicle treatment group was subjected to the identical experimental protocol, except that they received the same volume/weight of vehicle only.

#### 3.3.3. Morphometric Measurement of Infarct Volume and Edema

2,3,5-Triphenyltetrazolium chloride (TTC) staining was used to measure the cerebral infarction area. TTC is a reliable marker to highlight mitochondrial function, as well as stable indicator of ischemic areas for up to 3 days after ischemia [28,29]. Animals were decapitated after 22.5 h of reperfusion and their brains were carefully taken out. The brain slices were prepared along the coronal plane with a brain slicer for mice (World Precision Instruments, Inc., Sarasota, FL, USA) at 2 mm intervals, starting 1 mm from the frontal pole. TTC staining of the brain slices were performed by 60 min of incubation in a 2% solution of TTC at 37 °C followed by fixed-immersion with 10% neutral-buffered formalin. Computerized image analysis system (TDI Scope Eye 3.0, Samkyung Intl. Co., Seoul, Korea) was used to determine the infarction area (mm^2^) in each section. The total lesion volume (mm^3^) was calculated by summing the infarct area in each section (seven slices in all) and multiplying this by the distance between sections. Both contralateral and ipsilateral hemisphere areas were measured, and the differences between the two areas were used to calculate the edema volume. TTC and neutral-buffered formalin were purchased from the Sigma-Aldrich Co. (Natick, MA, USA). The significance of the differences between the total infarction volumes of the control (*n* = 10), MCI-186 (5 mg/kg, IV, *n* = 8), the selenoflavanone (40 mg/kg, IP, *n* = 8), and the flavanone (40 mg/kg, IP, *n* = 8) treated groups was determined by one way ANOVA followed by Student-Newman-Keuls test. * *p* < 0.05, ** *p* < 0.01, *** *p* < 0.001 compared with the control group. ^#^ *p* < 0.05, ## *p* < 0.01, ^###^ *p* < 0.001 compared with the flavanone treated group.

## 4. Conclusions

We synthesized selenoflavanones and flavanones using two steps, and evaluated their physicochemical properties and biological activities. The selenoflavanones showed lower polarity and higher lipophilicity than the corresponding flavanones, which suggests that they would be able to more easily penetrate the BBB. In our *in vitro* assay, hydrogen peroxide-induced cell death decreased with selenoflavanone treatment. Consequently, we confirmed the antioxidant effect and safety of the selenoflavanones. The total infarction volumes in the transient ischemia mouse model were significantly reduced by selenoflavanone treatment. Furthermore, the selenoflavanones resulted in more potent neuroprotective activity than the flavanones. Based on these observations, we anticipate that selenium substitution could be an effective approach for developing a neuroprotective agent.
